# Three isoforms of *Brugia malayi* *daf-16* activate a reporter gene in cultured HepG2 cells

**DOI:** 10.17912/micropub.biology.001823

**Published:** 2025-10-03

**Authors:** Cameron E Zehr, Alexius Folk, Katherine Stanford, Jenna Maiorelle, Kirsten Crossgrove

**Affiliations:** 1 Biology, University of Wisconsin–Whitewater, Whitewater, Wisconsin, United States; 2 Biological Sciences, University of Bergen, Bergen, Vestland, Norway; 3 University of Wisconsin–Madison, Madison, Wisconsin, United States

## Abstract

We hypothesize that infective stage molting in the parasitic nematode *Brugia malayi* is regulated by an ortholog of the *Caenorhabditis elegans daf-16* gene, similar to the role of *daf-16* in dauer formation and recovery. We confirmed the gene structure of three isoforms of *B. malayi daf-16 (Bma-daf-16)* and generated cell culture expression constructs for each. In luciferase assays using transfected HepG2 cells, all three isoforms activated a luciferase reporter gene regulated by six DAF-16 binding sites, although *Bma-* DAF-16a showed lower activation ability than *Bma-* DAF-16b and *Bma-* DAF-16c. These results support our hypothesis that *Bma-daf-16* functions similarly to *C. elegans daf-16* .

**Figure 1. Brugia malayi DAF-16 isoforms activate expression of a reporter gene.**
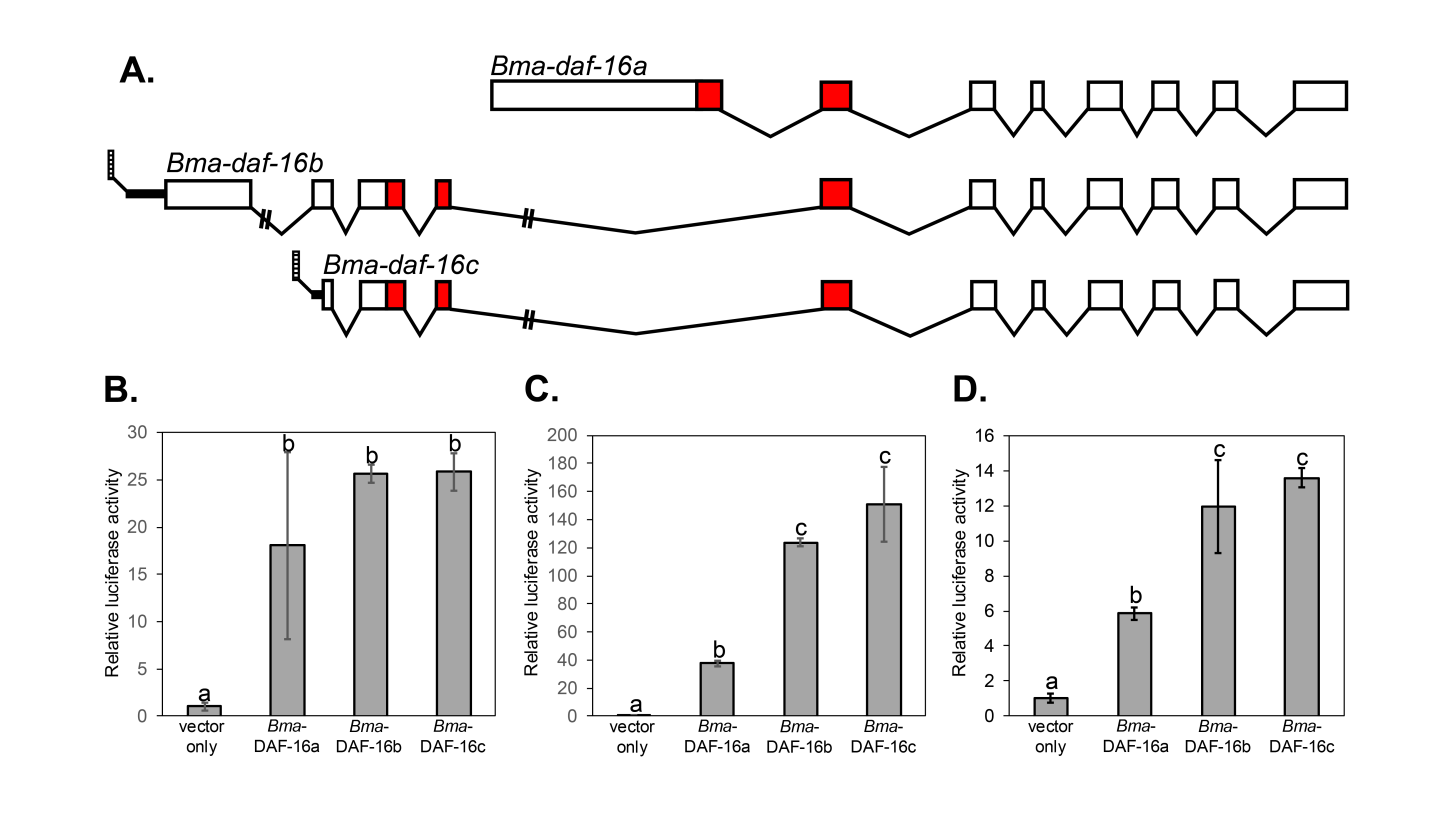
**A.** A schematic of the gene structure of three confirmed isoforms of the *Bma-daf-16* gene ( *Bm5392* in Wormbase version WS297) is shown (full sequence in extended data). Splicing was confirmed by RT-PCR using primers listed in Table 1 (methods). Boxes represent coding region exons, with red boxes indicating the coding region for the DNA binding domain(s). Introns are shown as lines below the exons and the 5' untranslated region for isoforms b and c is shown as a horizontal line with trans-splicing to spliced leader (striped box) indicated. Exon/intron sizes are shown to scale except where double lines intersect an intron. **B-D.** Three *Bma* -DAF-16 isoforms can activate transcription of a reporter gene regulated by DAF-16 binding elements (DBE). HepG2 cells in 24 well plates were transfected in triplicate with 250 ng p6XDBE- *luc* reporter, 50 ng pGL4.74 *Renilla* , and 200 ng of *HaloTag® CMV-neo* vector or *HaloTag® CMV-neo* vector expressing the indicated *Bma-* DAF-16 isoform. Relative luciferase activity is shown normalized to the average pHTC vector value. Error bars show standard deviation. Results were analyzed using one-way ANOVA. Conditions that do not share a letter show significant differences (p<0.05). The entire experiment was done in triplicate, and the results of each independent experiment are shown in Figures 1B, 1C, and 1D.

## Description

*Brugia malayi* , a parasitic nematode endemic to south and southeast Asia, causes lymphatic filariasis in humans due to the accumulation of adult parasites in the lymphatic system (Denham and McGreevy, 1977). The life cycle begins when adult females release microfilaria to the bloodstream, which are ingested by mosquitoes during a blood meal. Following two larval molts in the mosquito, the infective third larval (iL3) stage is transmitted to a new human host, molts twice to become an adult, and the cycle repeats (Nanduri and Kazura, 1989; Denham and McGreevy, 1977). According to the dauer hypothesis, the infective stage of parasitic nematodes is regulated similarly to the dauer larva l stage in the free-living model nematode *Caenorhabditis elegans* (Hotez et al., 1993; Bürglin et al., 1998; Crook, 2014).

The dauer stage in *C. elegans* is an alternative third larval stage which can form due to adverse conditions, such as overcrowding or lack of adequate nutrition (Cassada and Russell, 1975). Dauer larvae are non-feeding, stress resistant and metabolically inactive, and are induced to molt to the L4 stage when conditions improve (Cassada and Russell, 1975; Hu, 2007). The iL3 stage of parasitic nematodes is similar to dauer, in that they are also a metabolically inactive arrested stage which only molt to the L4 stage upon exposure to a new host environment (Crook, 2014). Dauer formation and recovery in *C. elegans* are regulated in part by the FOXO transcription factor DAF-16 , which is negatively regulated by an insulin/insulin-like growth factor signaling (IIS) pathway (Ogg et al., 1997; Lin et al., 1997; Murphy and Hu, 2013). Active DAF-16 promotes dauer formation, and when DAF-16 is inactive, dauer recovery can occur (Lee et al., 2001; Lin et al., 2001; Kwon et al., 2010; Aghayeva et al., 2021). We hypothesize that the infective stage of *B. malayi* is similarly regulated by a DAF-16 ortholog and IIS.

There are three predicted isoforms of the *B. malayi* *daf-16* gene ( *Bm5392* ) described in Wormbase version WS297 (Sternberg et al., 2024). We used Reverse Transcription-Polymerase Chain Reaction (RT-PCR) to confirm the predicted gene structure of all three *Bma-daf-16* isoforms ( Figure 1A ). In *C. elegans* , the *daf-16* gene uses alternative splicing and promoter usage to encode multiple protein isoforms that differ only in their N terminal sequences (Ogg et al., 1997; Lin et al., 1997; Kwon et al., 2010). Similar to *C. elegans* , the *B. malayi* isoforms have the same 3' coding region exons, but differ at their 5' ends. We used RT-PCR with the spliced leader (SL1) forward primer (Bektesh et al., 1988) and internal reverse primers to identify the 5' untranslated region for *Bma-daf-16b* (listed in Wormbase version WS297) and *Bma-daf-16c* (not currently shown in Wormbase version WS297).

We confirmed that *Bma-daf-16a* starts with a large 5' exon that includes the coding region for the N-terminus of the DNA binding domain (Casper et al., 2014). The structure of this isoform is similar to *C. elegans* isoform b (Wormbase version WS297 transcript R13H8.1a.1, protein isoform a), which encodes the DAF-16 b protein (Ogg et al., 1997; Lin et al., 2001; Kwon et al., 2010), and contains a DNA binding domain with a different N terminal section compared to all other *C. elegans* DAF-16 proteins (Ogg et al., 1997; Sternberg et al., 2024). *Bma-daf-16b* is most similar to *C. elegans* isoforms a1 and a2 (Wormbase version WS297 transcripts R13H8.1b.1 and R13H8.1c.1, protein isoforms b and c). These isoforms differ by only two amino acids, and the protein in *C. elegans* is generally referred to as DAF-16 a (Ogg et al., 1997; Lin et al., 2001; Kwon et al., 2010). *Bma-* DAF-16b and *Bma-* DAF-16c share the same DNA binding domain, which differs at the N terminus from *Bma-* DAF-16a. However, *Bma-daf-16c* , which begins in the second exon of *Bma-daf-16b* , encodes a protein with a truncated N terminus, making it most similar to the predicted *C. elegans* *daf-16* l and m isoforms (Wormbase version WS297 transcripts R13H8.1l.1 and R13H8.1m.1, protein isoforms l and m).

*C. elegans* DAF-16 acts as a transcription factor on multiple gene targets, including genes involved in dauer formation (Murphy, 2006). DAF-16 protein binds to a conserved DNA binding element (DBE, 5' TTGTTTAC 3'), both in vitro (Furuyama et al., 2000) and in vivo (Kumar et al., 2015). The *Bma* -DAF-16a DNA binding domain can bind to this DBE sequence in pulldown assays (Casper et al., 2014), suggesting that *Bma-* DAF-16 proteins have similar DNA binding specificity to *C. elegans* DAF-16 . *C. elegans* DAF-16 can activate a reporter gene containing an insulin-responsive element (IRE) in HepG2 cells (Nasrin et al., 2000). While the IRE sequence is not the same as the DBE, DAF-16 binds more strongly to the DBE than the IRE in mobility shift assays (Furuyama et al., 2000). A reporter gene construct containing six copies of the DBE can be activated in cultured NIH 3T3 cells by a DAF-16 /FOXO ortholog from the parasitic nematode *Ancylostoma caninum* (Gao et al., 2009). Since *Bma-* DAF-16 proteins can bind to the DBE, we hypothesized that they should similarly be able to activate a DBE regulated luciferase reporter gene.

To test the function of the three isoforms of *Bma-* DAF-16, we cloned the coding region of each isoform into the pHTC *HaloTag® CMV-neo* vector (Promega) to create eukaryotic cell culture expression constructs. Each isoform of *Bma-* DAF-16 increased expression of the firefly luciferase reporter gene compared to the vector control when these constructs, along with a p6XDBE- *luc* firefly luciferase reporter gene construct and pGL4.74[ *hRluc* /TK] *Renilla* luciferase expression construct, were used to transfect HepG2 cells ( Figure 1B- D). Further, *Bma-* DAF-16b and *Bma-* DAF-16c had significantly higher activation ability compared to *Bma-* DAF-16a in two out of three experiments ( Figure 1C, 1D). In the other experiment, there was high variation in the *Bma-* DAF-16a results ( Figure 1B ), which is likely the reason that no significant difference was observed.

We conclude that while activation ability may differ between isoforms, each of the three characterized isoforms of *Bma* -DAF-16 is separately able to activate a reporter gene containing six copies of the DBE in cultured HepG2 cells. This supports our hypothesis that *Bma-daf-16 (Bm5392)* is an ortholog of *C. elegans daf-16* with similar function, as predicted by the dauer hypothesis (Bürglin et al., 1998; Crook, 2014). We are currently using this cell culture system to investigate the ability of other *B. malayi* IIS genes to affect reporter gene activation by *Bma-* DAF-16.

## Methods

**Characterization of spliced forms of *Bma-daf-16* and generation of templates for cloning to expression vector**

Frozen *B. malayi* adult females and microfilaria were obtained from the NIH/NIAID Filariasis Research Reagents Resource Center (FR3; Michalski et al., 2011) and RNA was isolated using TriReagent (Ambion). DNA was removed using RNase-free TurboDNAse (Invitrogen) and first strand cDNA was synthesized using a High Capacity Reverse Transcription kit (Applied Biosystems) with random hexamer primers. Amplitaq Gold DNA polymerase (Applied Biosystems), with 1.5 mM MgCl _2_ and 0.4 mM dNTPs, or Amplitaq Gold 360 master mix (Applied Biosystems/ThermoFisher), were used to amplify cDNA with 20 pmoles of forward and reverse primers (Table 1) with varying cycling conditions (95°C 5 min; 35-40 cycles of 95°C 15-60 sec, varying annealing temperatures 30-60 sec, 72°C 1-2 min; 72°C 7-10 min; 4°C hold). PCR products were gel or column purified (Wizard® SV Gel and PCR Clean-Up System, Promega) and either directly sequenced or ligated to the pGEM®-T Easy vector (Promega) after which individual clones were prepared (Promega PureYield ^TM^ Miniprep System) and sequenced. Sequencing reactions for *Bma-daf-16a* and *Bma-daf-16b* were prepared with BigDye v3.1 mix (Applied Biosystems) followed by cleanup and capillary gel electrophoresis at the University of Wisconsin Biotechnology Center (UWBC). *Bma-daf-16c* DNA was sequenced by Eurofins Genomics.

**Table:** 

Primer Name	Primer Sequence	Primer Use
** Primers used to amplify *Bma-daf-16a* to confirm gene structure **		
*Bma-daf-16-3*	5'ATGGAAGCAAGAGATTCAGAG3'	forward primer for amplifying 5' section of *Bma-daf-16a,* hybridizes at 1-21 of coding region
*Bma-daf-16-30*	5'CTTCAGGATTTTCCGGATCA3'	reverse primer for amplifying 5' section of *Bma-daf-16a,* hybridizes at 936-955 of coding region
*Bma-daf-16-5*	5'TGATCCGGAAAATCCTGAAG3'	forward primer for amplifying middle section of *Bma-daf-16a,* hybridizes at 936-955 of coding region
*Bma-daf-16-8*	5'GATGGCGATACTCGTGATGA3'	reverse primer for amplifying middle section of *Bma-daf-16a* , hybridizes at 1552-1571 of coding region
*Bma-daf-16-1*	5'TCGAATTTTGAACCTTTCCG3'	forward primer for amplifying 3' section of *Bma-daf-16a* , hybridizes at 1495-1514 of coding region
*Bma-daf-16-4*	5'CTAAATATTGTCGAAACTAAGCTG3'	reverse primer amplifying 3' section of *Bma-daf-16a,* hybridizes at 2173-2196 (3' end of coding region)
** Primers used to amplify *Bma-daf-16b* to confirm gene structure **		
SL1	5'GGTTTAATTACCCAAGTTTGAG3'	forward primer for amplifying 5' section of *Bma-daf-16b* , hybridizes to spliced leader
*Bma-daf-16-23*	5'CCTCTTCCGGAATTTGTTCA3'	reverse primer for amplifying 5' section of *Bma-daf-16b,* hybridizes at 408-427 of coding region
*Bma-daf-16-28*	5'TGAACAAATTCCGGAAGAGG3'	forward primer for amplifying middle section of *Bma-daf-16b,* hybridizes at 408-427 of coding region
*Bma-daf-16-8*	5'GATGGCGATACTCGTGATGA3'	reverse primer for amplifying middle section of *Bma-daf-16b* , hybridizes at 1177-1196 of coding region
** Primers used to amplify *Bma-daf-16c* to confirm gene structure **		
SL1	5'GGTTTAATTACCCAAGTTTGAG3'	forward primer for amplifying 5' end of *Bma-daf-16c* , hybridizes to spliced leader
*Bma-daf-16-89*	5'AGCAACACTCGAGTCGGATG3'	reverse primer for ampifying 5' end of *Bma-daf-16c,* hybridizes at 107-126 of *Bma-daf-16c* coding region
*Bma-daf-16-90*	5'TAGCCGCTTTTCAGGTGAAC3'	reverse primer for ampifying 5' end of *Bma-daf-16c,* hybridizes at 209-228 of *Bma-daf-16c* coding region
** Primers used for cloning of *Bma-daf-16a* to pHTC Halotag vector **		
*Bma-daf-16-25*	5'GAT **GCTAGC** ATGGAAGCAAGAGATTCAGAG3'	forward primer, hybridizes to 5' end of coding region and contains *Nhe* I site (boldface)
*Bma-daf-16-27*	5'GAT **CTCGAG** AATATTGTCGAAACTAAGCTGACTAC3'	reverse primer, hybridizes to 3' end of coding region before stop codon and contains *Xho* I site (boldface)
** Primers used for cloning of *Bma-daf-16b* to pHTC Halotag vector **		
*Bma-daf-16-26*	5'GAT **GCTAGC** ATGTTATCAGCATCTTCTGGTAATTG3'	forward primer, hybridizes to 5' end of coding region and contains *Nhe* I site (boldface)
*Bma-daf-16-27*	5'GAT **CTCGAG** AATATTGTCGAAACTAAGCTGACTAC3'	reverse primer, hybridizes to 3' end of coding region before stop codon and contains *Xho* I site (boldface)
** Primers used for cloning of *Bma-daf-16c* to pHTC Halotag vector **		
*Bma-daf-16-91*	5'taatacgactcactatagggATGGGTTCGCCGGAAAGTG3'	forward primer for HiFi assembly, hybridizes to 5' end of coding region, vector overlap shown as lowercase
*Bma-daf-16-92*	5'acagatcctcagtggttggctcAATATTGTCGAAACTAAGCTGACTAC3'	reverse primer for HiFi assembly, hybridizes to 3' end of coding region before stop codon, vector overlap shown as lowercase

**Table 1. Primers used in this study.**

**Generation of expression constructs**

*Bma-daf-16a* and *Bma-daf-16b*

*Bma-daf-16a* and *Bma-daf-16b* were cloned in sections due to difficulty in amplifying the entire coding region from *B. malayi* cDNA. Overlapping fragments were gel purified and combined in PCR reactions in which 15 cycles were conducted using just the PCR products, followed by 20 cycles including primers that hybridize to the 5' and 3' ends of the overlapped product (Table 1). Full length cDNA was generated with *Nhe* I and *Xho* I restriction enzyme sites on the ends. This product was gel purified and cloned to the pGEM®-T Easy vector (Promega). Individual clones were prepared and sequenced. After confirming the sequence, one clone was digested with *Xho* I and *Nhe* I and the gel purified product was ligated to the *pHTC HaloTag® CMV-neo* vector (Promega) at the *Xho* I and *Nhe* I sites to allow expression of *Bma-* DAF-16 with a C-terminal Halotag fusion. JM109 cells (Promega) were transformed with the ligation mix and individual colonies were prepared and sequenced as described above. When the *Bma-daf-16b* product was cloned to pGEM®-T Easy and cut with *Nhe* l and *Xho* I, an extra product was generated. Sequencing showed that the splicing at the 5' end was different than what was listed in Wormbase at the time (version WS244) and contained an *Xho* I site. Partial digestion was used to purify a product in which the internal *Xho* I site was not cut and this was used for cloning to *pHTC HaloTag® CMV-neo* .

*Bma-daf-16c*

The *Bma-daf-16c* expression construct was generated using HiFi assembly (New England Biolabs). Specifically, the coding region of *Bma-daf-16c* was amplified using Q5® High-Fidelity DNA Polymerase (New England Biolabs) using the pHTC/ *Bma-daf-16b* expression construct as a template. The product was gel purified and combined with *pHTC HaloTag® CMV-neo* vector cut with *Nhe* I and *Xho* I using Hi-Fi DNA Assembly mix (New England Biolabs) and used to transform NEB® 5-alpha *E. coli* cells (New England Biolabs). Plasmid DNA from transformed *E. coli* cells was isolated using the PureYield ^TM^ Plasmid DNA Miniprep System (Promega), and sequences were confirmed using whole-plasmid sequencing (Eurofins Genomics).

**HepG2 cell maintenance**

HepG2 cells (ATCC) were cultured in EMEM/10%FBS (changed every 2-4 days) at 37°C/5%CO _2_ . Cells were split using trypsin when cells reached 70-80% confluence. Since the cells were prone to clumping, as previously observed for HepG2 (ATCC), they were exposed to trypsin for up to 20 minutes and mechanically disrupted by pipetting up and down prior to resuspension in media.

**Transfection of HepG2 cells and measurement of reporter gene activity**

Prior to transfection, HepG2 cells were plated on 24 well plates in 500 µL EMEM/10%FBS with antibiotics and antimycotics. Once cells reached approximately 50-70% confluence, media was replaced with EMEM/10%FBS without antibiotics or antimycotics, and cells were transfected with 250 ng p6XDBE- *luc* (Gao et al., 2009; firefly luciferase reporter with six DAF-16 DBE, kindly provided by Dr. Xin Gao, Washington University, and Dr. John Hawdon, George Washington University, a gift of Professor B.M. Burgering, University Medical Centre Utrecht, Utrecht, The Netherlands), 50 ng pGL4.74[ *hRluc* /TK] vector (Promega), and 200 ng of the appropriate pHTC/ *Bma-daf-16* expression construct or vector alone. All plasmid DNA for transfections was prepared using the PureYield ^TM^ Plasmid DNA Midiprep System (Promega). Triplicate wells were done for each isoform and the vector only control, as well as a no transfection control. Cells were transfected according to the ViaFect ^TM^ Transfection Reagent Protocol (Promega), using 6 µL transfection reagent per 1 µg of DNA. After 24 hours, media was removed from plates, wells were washed using 500 µL 1XPBS, and cells were incubated for 15 minutes at room temperature on a nutator in 100 µL 1X passive lysis buffer (Promega). 20 µL of each cell lysate was pipetted into each of two duplicate wells on a 96 well white bottom plate (Thermo Fisher Scientific), and luciferase reporter activity was measured using a Dual Luciferase Assay (Promega), with 50 µL injector volumes, using a Glomax luminometer (Promega). The average background (no transfection control) values were subtracted from all other readings, firefly luciferase values were divided by *Renilla* luciferase values to control for transfection efficiency, duplicate wells were averaged, and those values were divided by the average pHTC ‘vector only' value to give final results in relative light units. The entire experiment was performed three times.

**Statistics**

Averages of duplicate luciferase assay results, given in relative light units, for each transfected well were treated as individual data points. Differences between transfection conditions were tested using one-way ANOVA with Tukey's HSD (α = 0.05) at https://www.statskingdom.com/180Anova1way.html (accessed on 07/25/25). The number of data points per transfection condition was always three.

## Reagents

**Table:** 

**Plasmid name**	**Construct / Function**	**Location**
pGEM®-T Easy Vector	Vector for cloning products amplified by *Taq* polymerase	Promega
pgBmAF01	Vector with *Bma-daf-16b* 5' end cloned in for sequencing	This study
pgBmCZ03	Vector with *Bma-daf-16c* 5' end cloned in for sequencing	This study
pgBmCZ04	Vector with *Bma-daf-16c* 5' end cloned in for sequencing	This study
p6XDBE- *luc*	*6XDBE::minP::luc* / Firefly luciferase expression construct containing 6 binding sites for DAF-16	Kindly provided by Dr. Xin Gao, Washington University, and Dr. John Hawdon, George Washington University, a gift of Professor B.M. Burgering, University Medical Centre Utrecht, Utrecht, The Netherlands
pGL4.74[ *hRluc* /TK]	*HSV-TK::hRluc* / *Renilla* luciferase expression construct to control for transfection efficiency	Promega
pHTC Halotag® CMV-neo	*CMV::HaloTag* / Vector for generating expression constructs, and vector control for cell culture	Promega
phBmLS01	*CMV::Bma-daf-16a::HaloTag* / Eukaryotic expression construct for *Bma-daf-16* isoform a	This study
phBmJM01	*CMV::Bma-daf-16b::HaloTag* / Eukaryotic expression construct for *Bma-daf-16* isoform b	This study
phBmCZ01	*CMV::Bma-daf-16c::HaloTag* / Eukaryotic expression construct for *Bma-daf-16* isoform c	This study

## Data Availability

Description: DNA sequences of confirmed Bma-daf-16 isoforms. Resource Type: Text. DOI: https://doi.org/10.22002/myhrh-ftm83
